# ﻿Phylogenetic relationships among the species of the Cameroonian endemic freshwater crab genus *Louisea* Cumberlidge, 1994 (Crustacea, Brachyura, Potamonautidae), with notes on intraspecific morphological variation within two threatened species

**DOI:** 10.3897/zookeys.1122.85791

**Published:** 2022-09-26

**Authors:** Pierre A. Mvogo Ndongo, Thomas von Rintelen, Paul F. Clark, Adnan Shahdadi, Carine Rosine Tchietchui, Neil Cumberlidge

**Affiliations:** 1 Département de Gestion des Écosystèmes Aquatiques, Institut des Sciences Halieutiques, Université de Douala à Yabassi, PO. Box. 7236 Douala-Bassa, Cameroon Museum für Naturkunde, Leibniz Institute for Evolution and Biodiversity Science Berlin Germany; 2 Museum für Naturkunde, Leibniz Institute for Evolution and Biodiversity Science, Invalidenstrasse 43, 10115 Berlin, Germany Université de Douala à Yabass Douala-Bassa Cameroon; 3 Department of Life Sciences, The Natural History Museum, London, SW7 5BD, UK Department of Life Sciences, The Natural History Museum London United Kingdom; 4 Department of Marine Biology, Faculty of Marine Sciences and Technology, University of Hormozgan, Bandar Abbas, Iran University of Hormozgan Bandar Abbas Iran; 5 Zoology Unit, Laboratory of Biology and Physiology of Animal Organisms, Faculty of Science, University of Douala, PO Box 24157 Douala, Cameroon University of Douala Douala Cameroon; 6 Department of Biology, Northern Michigan University, Marquette, MI 49855-5376, USA Northern Michigan University Marquette United States of America

**Keywords:** Decapoda, morphotypes, Nkongsamba, Potamoidea, species boundaries, Yabassi

## Abstract

*Louisea* Cumberlidge, 1994 (Crustacea, Brachyura, Potamonautidae) currently includes four endemic Cameroonian freshwater crab species whose phylogenetic relationships were previously unresolved. In the present study, phylogenetic analyses are carried out involving three mtDNA loci (COI, 12S rRNA, and 16S rRNA). The COI locus revealed divergence times of 5.6 million years ago (myr) for when *L.balssi* (Bott, 1959) diverged from *L.edeaensis* (Bott, 1969); 4.1 myr for when *L.edeaensis* diverged from *L.yabassi* Mvogo Ndongo, von Rintelen & Cumberlidge, 2019; and 2.48 myr for when the later species diverged from *L.nkongsamba* Mvogo Ndongo, von Rintelen & Cumberlidge, 2019. Three genetic lineages were found within *L.nkongsamba* that are supported by uncorrected *p*-distances and the haplotype network. Morphological variation in some taxonomically important characters was found within both *L.nkongsamba* and *L.yabassi*. No correlation, however, was found between the morphotypes within these species and the uncovered genetic lineages. Recognition of species boundaries and of subpopulations of species will prove valuable when making informed conservation decisions as part of the development of species action plans for these rare and threatened freshwater crabs.

## ﻿Introduction

*Louisea* Cumberlidge, 1994 (Crustacea, Brachyura, Potamonautidae) is endemic to remote Cameroonian forested ecosystems and currently includes four freshwater crab species: *L.balssi* (Bott, 1959), *L.edeaensis* (Bott, 1969), *L.nkongsamba* Mvogo Ndongo, von Rintelen & Cumberlidge, 2019, and *L.yabassi* Mvogo Ndongo, von Rintelen & Cumberlidge, 2019. *Louiseabalssi* and *L.edeaensis* have been revised recently based on new material collected in Cameroon (Mvogo Ndongo et al. 2017a, 2018, 2019), while *L.nkongsamba* and *L.yabassi* were recently discovered (Mvogo Ndongo et al. 2019). Other works on Cameroonian freshwater crabs have mainly focused on their taxonomy, phylogenetic relationships, or conservation (Cumberlidge 1999; Daniels et al. 2015; Mvogo Ndongo et al. 2017a, 2017b, 2017c, 2018, 2019, 2020, 2021a; Cumberlidge and Daniels 2022). This is the first study, however, that includes both morphological and molecular data from all known *Louisea* species. The present work also includes new collections of two *Louisea* species from the forested sites in southwestern Cameroon: *L.yabassi* from the Ebo Forest, and *L.nkongsamba* from the Nlonako Ecological Reserve (Mvogo Ndongo et al. 2019, 2021b). These populations are compared with those of *L.balssi* from Kumba and Mount Manengouba (Cumberlidge 1994, 1999; Mvogo Ndongo et al. 2017a, 2017c, 2018, 2019), and of *L.edeaensis* from Yaounde, Edea, and Lake Ossa (Cumberlidge 1994, 1999; Mvogo Ndongo et al. 2017a, 2017c, 2019).

The aim of the present work is to evaluate the phylogenetic relationships within *Louisea* and to estimate the genetic distance between the species using molecular data. Intraspecific variation of some important taxonomic characters within two newly discovered species is also assessed in order to better identify species boundaries within *Louisea*. Accurate species delimitation is necessary for understanding levels of biodiversity, and for adopting effective conservation and sustainable management strategies (Cornetti et al. 2015). The results from this study will be helpful in developing action plans aimed at the conservation of these rare, threatened, and endemic Cameroonian freshwater crab species.

## ﻿Materials and methods

### ﻿Sample collection

**Figure 1.**
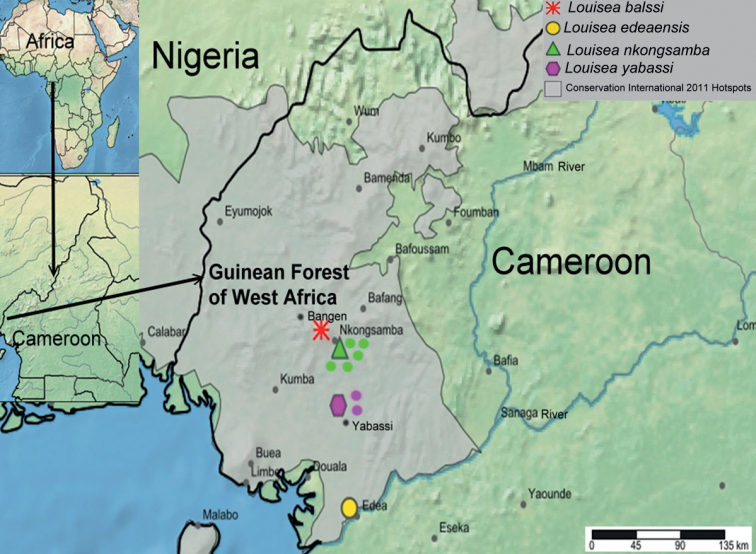
Map of Cameroon showing collection sites of *Louisea* species. *Louiseankongsamba*: type locality (green triangle), new localities (green circles); *Louiseayabassi*: type locality (purple hexagon), new localities (purple circles).

Four *Louisea* species were collected from four different locations in southwestern Cameroon between 2015 and 2021 (Fig. 1). The species were identified by following Cumberlidge (1994, 1999) and Mvogo Ndongo et al. (2019). Eight specimens of *L.balssi* were collected from 1,958 m a.s.l., Mount Manengouba; 30 specimens of *L.edeaensis* from 90 m a.s.l., Bedimet Island, Lake Ossa; 50 specimens of *L.nkongsamba* from 1000–1400 m a.s.l., Mount Nlonako; and 35 specimens of *L.yabassi* from up to 300 m a.s.l., the Ebo Forest near Yabassi. Specimens of *L.nkongsamba* and *L.yabassi* were studied to clarify intraspecific morphological variation within each species. Specimens were measured; their gender and life stage (juvenile, subadult, and adult) recorded; and their habitat preferences noted. Most of the crabs were released into their natural habitat after recording all relevant morphological data. Only a few whole adult specimens (males and females), as well as one of the walking legs was removed from each of the other selected specimens were preserved in ethanol for further morphological descriptions and molecular analyses. The newly collected specimens were deposited either in the Museum für Naturkunde, Berlin, Germany (**ZMB**), or in the Unity of Taxonomy, Production and Sustainable Management of Aquatic Animals, Department of Management of Aquatic Ecosystems, Institut des Sciences Halieutiques, University of Douala, Cameroon (**LABO-PASMAT**).

### ﻿Morphological analyses

Descriptive morphometrics of *L.edeaensis* and *L.balssi* specimens are given in Mvogo Ndongo et al. (2019: tables 2 and 3, respectively). Measurements (in mm) of the carapace of all the specimens were made with digital callipers. Characters of the carapace, thoracic sternum, chelipeds, and mandibles were examined in detail. The terminology used follows Cumberlidge (1999), and the classification by Cumberlidge and Daniels (2022). Images of the body parts were taken using a Leica microscope (model Z16A POA), and the LAS v. 4 and Helicon Focus v. 6.7.1 software. Post processing was undertaken using Adobe Photoshop CC5. Specimens were sorted according to their stage of development into juveniles, subadults, and adults. Furthermore, the maturity of adults was deciphered by identifying specimens that had undergone the pubertal moult from subadult to adult. The pubertal moult was determined by examining the degree of development of the pleon of a series of juvenile, subadult and adult females. The pleon of juvenile females is undeveloped and resembles the slim pleon of juvenile males; the pleon of subadults is significantly widened and partially covers the thoracic sternum. In comparison, the pleon of adult females is conspicuously enlarged and rounded such that its lateral margins overlap the coxae of the pereiopods, and the telson covers thoracic sternites 1 and 2. The lower limit of the range for the pubertal moult was judged as the CW of the largest non-adult female, while the upper limit of the pubertal moult was the CW of the smallest adult female.

### ﻿Molecular analyses

Genomic DNA was extracted from a tissue sample of up to 25 mg cut from the pereiopod muscle of 70% ethanol-preserved specimens using the Qiagen DNeasy Blood & Tissue kit following the manufacturer’s instructions. Polymerase chain reaction (PCR) was used to amplify three mitochondrial gene fragments: a ~638 bp region of the 16S ribosomal RNA gene (16S rRNA) using primers 16L29 and 16HLeu (Schubart 2009); a ~594 bp region of the 12S ribosomal RNA gene (12S rRNA) using primers 12L4 and 12H2 (Schubart et al. 2006); and a 648 bp region of the protein-coding mitochondrial gene, cytochrome oxidase subunit I gene (COI) using primers LCO-1490 and HCO-2198 (Folmer et al. 1994). PCR was performed in 25 μl volumes containing 1× Taq buffer, 1.5 mM MgCl_2_, 200 μM each dNTP, 1 U Taq polymerase, ~50–100 mg DNA and ddH_2_O up to volume. After an initial denaturation step of 4 min at 94 °C, cycling conditions were 35 cycles at 94 °C for 30 s, 45 °C for 60 s, and 72 °C for 90 s, with a final elongation step of 5 min at 72 °C. The same primers were used in PCR and sequencing. PCR products were sent to Macrogen Europe for purification and cycle sequencing of both strands of each gene. The sequences obtained were proofread manually using Chromas Lite (v. 2.1.1) (Technelysium Pty Ltd, Queensland, Australia) and aligned with ClustalW (Thompson et al. 1994) implemented in BioEdit 7.0.5 (Hall 1999). New sequences were submitted to the National Center for Biotechnology Information and are available from GenBank under the accession numbers in Table 1. Results from these genes were concatenated into a single alignment, which was then converted into a Nexus file with FaBox (Villesen 2007).

**Table 1.** Details of mtDNA markers used in the present study for *Louisea* species and outgroup species. Nl = Nlonako; Here = sequence available in the present study; * = Mvogo Ndongo et al. 2019; ** = Mvogo Ndongo et al. 2017c.

Species and sample number	Locality in Cameroon	Population number	Morphotypes (see Tables 3, 4)	Museum/extraction number	GenBank accession number		
					COI	12S rRNA	16S rRNA
*L.nkongsamba* (1)	Nlonako, Engugue1382	Population 1	Nl Morphotype 1	ZMB-X21	OP122926	OP133321	OP133281
*L.nkongsamba* (2)	Nlonako, NgaltongueS1	Population 1	Nl Morphotype 1	ZMB-X26	OP122931	OP133326	OP133286
*L.nkongsamba* (3)	Nlonako, NgaltongueS1	Population 1	Nl Morphotype 1	ZMB-X27	OP122932	OP133327	OP133287
*L.nkongsamba* (4)	Nlonako, NgaltongueS1	Population 1	Nl Morphotype 1	ZMB-X28	OP122933	OP133328	OP133288
*L.nkongsamba* (5)	Nlonako, NgaltongueS1	Population 1	Nl Morphotype 1	ZMB-X29	OP122934	OP133329	OP133289
*L.nkongsamba* (6)	Nlonako Engugue1462	Population 1	Nl Morphotype 1	ZMB-X31	OP122936	OP133331	OP133291
*L.nkongsamba* (7)	Nlonako, NgaltongueS2	Population 1	Nl Morphotype 1	ZMB-X36	OP122941	OP133336	OP133296
*L.nkongsamba* (8)	Nlonako, NgaltongueS2	Population 1	Nl Morphotype 1	ZMB-X37	OP122942	OP133337	OP133297
*L.nkongsamba* (9)	Nlonako, NgaltongueS2	Population 1	Nl Morphotype 1	ZMB-X38	OP122943	OP133338	OP133298
*L.nkongsamba* (10)	Nlonako, NgaltongueS2	Population 1	Nl Morphotype 1	ZMB-X39	OP122944	OP133339	OP133299
*L.nkongsamba* (11)	Nlonako, Eyimba	Population 1	Nl Morphotype 1	ZMB-X41	OP122946	OP133341	OP133301
*L.nkongsamba* (12)	Nlonako, Nguengue	Population 1	Nl Morphotype 1	ZMB-X46	OP122951	OP133346	OP133306
*L.nkongsamba* (13)	Nlonako, Nguengue	Population 1	Nl Morphotype 1	ZMB-X47	OP122952	OP133347	OP133307
*L.nkongsamba* (14)	Nlonako, Nguengue	Population 1	Nl Morphotype 1	ZMB-X48	OP122953	OP133348	OP133308
*L.nkongsamba* (15)	Nlonako, Engugue1382	Population 2	Nl Morphotype 1	ZMB-X22	OP122927	OP133322	OP133282
*L.nkongsamba* (16)	Nlonako, Engugue1382	Population 2	Nl Morphotype 1	ZMB-X23	OP122928	OP133323	OP133283
*L.nkongsamba* (17)	Nlonako, Engugue1382	Population 2	Nl Morphotype 1	ZMB-X24	OP122929	OP133324	OP133284
*L.nkongsamba* (18)	Nlonako, NgaltongueS1	Population 2	Nl Morphotype 1	ZMB-X30	OP122935	OP133330	OP133290
*L.nkongsamba* (19)	Nlonako Engugue1462	Population 2	Nl Morphotype 2	ZMB-X32	OP122937	OP133332	OP133292
*L.nkongsamba* (20)	Nlonako Engugue1462	Population 2	Nl Morphotype 2	ZMB-X33	OP122938	OP133333	OP133293
*L.nkongsamba* (21)	Nlonako Engugue1462	Population 2	Nl Morphotype 2	ZMB-X34	OP122939	OP133334	OP133294
*L.nkongsamba* (22)	Nlonako, Eyimba	Population 2	Nl Morphotype 1	ZMB-X42	OP122947	OP133342	OP133302
*L.nkongsamba* (23)	Nlonako, Eyimba	Population 2	Nl Morphotype 1	ZMB-X43	OP122948	OP133343	OP133303
*L.nkongsamba* (24)	Nlonako, Eyimba	Population 2	Nl Morphotype 1	ZMB-X44	OP122949	OP133344	OP133304
*L.nkongsamba* (25)	Nlonako, Nguengue	Population 2	Nl Morphotype 1	ZMB-X49	OP122954	OP133349	OP133309
*L.nkongsamba* (26)	Nlonako, Nguengue	Population 2	Nl Morphotype 1	ZMB-X50	OP122955	OP133350	OP133310
*L.nkongsamba* (27)	Nlonako, Engugue1382	Population 3	Nl Morphotype 1	ZMB-X25	OP122930	OP133325	OP133285
*L.nkongsamba* (28)	Nlonako Engugue1462	Population 3	Nl Morphotype 1	ZMB-X35	OP122940	OP133335	OP133295
*L.nkongsamba* (29)	Nlonako, NgaltongueS2	Population 3	Nl Morphotype 1	ZMB-X40	OP122945	OP133340	OP133300
*L.nkongsamba* (30)	Nlonako, Eyimba	Population 3	Nl Morphotype 1	ZMB-X45	OP122950	OP133345	OP133305
*L.yabassi* (31)	Eboforest Stream no. 1	Population 1	Ebo Morphotype 1	ZMB-X11	OP122956	OP133351	OP133311
*L.yabassi* (32)	Eboforest Stream no. 1	Population 1	Ebo Morphotype 1	ZMB-X12	OP122957	OP133352	OP133312
*L.yabassi* (33)	Eboforest Stream no. 1	Population 1	Ebo Morphotype 1	ZMB-X13	OP122958	OP133353	OP133313
*L.yabassi* (34)	Eboforest Stream no. 1	Population 1	Ebo Morphotype 1	ZMB-X14	OP122959	OP133354	OP133314
*L.yabassi* (35)	Eboforest Stream no. 1	Population 1	Ebo Morphotype 1	ZMB-X15	OP122960	OP133355	OP133315
*L.yabassi* (36)	Eboforest Stream no. 2	Population 2	Ebo Morphotype 2	ZMB-X16	OP122961	OP133356	OP133316
*L.yabassi* (37)	Eboforest Stream no. 2	Population 2	Ebo Morphotype 2	ZMB-X17	OP122962	OP133357	OP133317
*L.yabassi* (38)	Eboforest Stream no. 2	Population 2	Ebo Morphotype 2	ZMB-X18	OP122963	OP133358	OP133318
*L.yabassi* (39)	Eboforest Stream no. 2	Population 2	Ebo Morphotype 2	ZMB-X19	OP122964	OP133359	OP133319
*L.yabassi* (40)	Eboforest Stream no. 2	Population 2	Ebo Morphotype 2	ZMB-X20	OP122965	OP133360	OP133320
*L.edeaensis*	Lake Ossa, Bedimet Island	Population 1	—	ZMB Crust 30335	MN188068.1*	—	MN217395*
*L.edeaensis*	Lake Ossa, Bedimet Island	Population 1	—	T351-30	KY964474.1**	KY964479**	KY964472**
*L.edeaensis*	Lake Ossa, Bedimet Island	Population 1	—	ZMB_Crust 26930	KY964473.1**	KY964478**	—
*L.balssi*	Manengouba, stream	Population 1	—	ZMB Crust 30319	MN188071.1*	MN217385*	MN217392*
*L.balssi*	Manengouba, stream	Population 1	—	ZMB Crust.29628	MN188070.1*	MN217384*	MN217391*
*Potamonemusman*	Bakossi National Park	Population 1	—	ZMB Crust 30327	MN188067.1*	MN217390*	MN217398*
*Bueamundemba*	Korup National Park	Population 1	—	ZMB Crust 30321	MN188069.1*	MN217388*	MN217396*

### ﻿Phylogeographic investigations

The COI mitochondrial gene employed here is relatively variable and is commonly used for population genetics, and more recently also for faunal species identification using the barcoding approach (Hebert et al. 2003). This was useful for the examination of the population structure of *L.nkongsamba*, which provides evidence for genetic substructure among the sampling sites in Nlonako Ecological Reserve. These data are critical for the investigation of the historical connectivity among populations of *Louisea* species and are useful for the implementation of the future management of genetic diversity.

Maximum parsimony genotype networks (Templeton et al. 1992) were built with the software PopArt (Leigh and Bryant 2015) in order to graphically depict the genetic distances between mitochondrial genotypes. Haplotype and nucleotide diversities were used to compare genetic diversities among the sampling sites in terms of the number of haplotypes and the genetic distances of these haplotypes. Phylogeographic investigations have been successfully used by several researchers to determine connectivity among populations of other endemic crab species, e.g., *Sesarmafossarum* Schubart, Reimer, Diesel & Türkay, 1997, from the Cockpit Country, Jamaica (see Stemmer and Schubart 2016).

### ﻿Phylogenetic investigations

The mitochondrial genes (COI, 12S rRNA, 16S rRNA) were used to identify the species boundaries, to examine the evolutionary origins and the relationships within *Louisea* species, and to determine whether morphological and ecological similarities between species are based on convergence or common ancestry. Here two methods of phylogenetic inference were applied to the data set: maximum likelihood (ML) using the software PAUP*, and Bayesian inference (BI) as implemented in MrBayes (v. 3.3; Huelsenbeck and Ronquist 2001) (see Mvogo Ndongo et al. 2017b, 2017c; Fratini et al. 2005). The best evolutionary model was determined with jModeltest v. 2.1.7 (Darriba et al. 2012) based on the Akaike Information Criterion (Posada and Buckley 2004) and resulted in the GTR+I+G (COI), GTR+G (16S rRNA) and HKY+G (12S rRNA) models. ML tree was obtained for each alignment with 1000 bootstrap pseudoreplicates. BI was performed to infer phylogeny by using MrBayes v. 3.2.2 (Huelsenbeck and Ronquist 2001). The Markov Chain Monte Carlo was run with four independent chains for 10,000,000 generations, samplefreq = 500, and burnin = 10,001. Analyses were conducted separately to test for topology congruence.

A total of 138 DNA sequences were obtained, 46 sequences each of COI, 16S rRNA, and 12S rRNA (Table 1). ML and BI trees were constructed for individual gene. The relative tree presented here for ML topology has been reconstructed from the concatenation of the three partial loci (COI, 16S rRNA, and 12S rRNA) into a single alignment, which was then converted into a Nexus file with FaBox. This tree includes *L.balssi*, *L.edeaensis*, *L.nkongsamba*, and *L.yabassi* as the in-group, and *Potamonemusman* Mvogo Ndongo, von Rintelen & Cumberlidge, 2021a and *Bueamundemba* Mvogo Ndongo, von Rintelen & Cumberlidge in Mvogo Ndongo, von Rintelen, Tomedi-Tabi & Cumberlidge, 2020 as the out-group species.

To estimate clade divergence times based on the COI gene, a Bayesian analysis with the software BEAST v. 2.6.2 (Bouckaert et al. 2019) was conducted using a strict clock model (Yule Model) with a rate of evolution for the COI of 2.33% per million years (my) (10% SD) (following Schubart et al. 1998). Markov chains for 10 million generations were undertaken, sampling every 1000^th^ iteration and discarding the first 25% as burn-in. Overall, 7500 trees were obtained, and these trees were used to calculate the maximum clade credibility tree in TreeAnnotator v. 1.6.1 (part of the BEAST package). The uncorrected *p*-distances (%) was calculated in MEGA 7 (Kumar et al. 2016).

#### Abbreviations used

**a.s.l.** above sea level;

**CW** carapace width measured at widest point;

**CL** carapace length measured along medial line from anterior to posterior margin;

**CH** carapace height measured at maximum height of cephalothorax;

**FW** front width measured along anterior frontal margin between inner angles of orbits;

**myr** million years ago;

**PAMN** Pierre A. Mvogo Ndongo;

**S2/3** male sternal sulcus between thoracic sternites 2 and 3;

**S3/4** male sternal sulcus between thoracic sternites 3 and 4.

## ﻿Results

### ﻿Morphological analyses

Morphometric measurements of *L.yabassi* and *L.nkongsamba* populations are provided in Table 2. The adult size range of *L.yabassi*, based on male and female specimens from the two populations, was determined to be between CW 16.5 and CW 24.0 mm. Subadults of *L.yabassi* ranged from CW 11.0 mm to CW 15.5 mm, whereas juveniles of this species were CW 10.0 mm or less. The adult size range of *L.nkongsamba*, based on male and female specimens from four of the six sites, was between CW 15.8 mm and CW 20.0 mm. Subadults of *L.nkongsamba* ranged from CW 11.5 mm to CW 14.4 mm (two populations, PAMN 02.12.19 and PAMN 10.12.19, consisted entirely of subadults), whereas juveniles of this species measured CW 10.0 mm or less. No major differences were found between the carapace proportions (CW/FW, CL/FW, and CH/FW) of any of the populations of these two species, and these proportions were virtually identical in all cases (Table 2). The difference between the adult size range of *L.yabassi* and *L.nkongsamba* is minor, with the former species growing up to CW 24 mm and the latter species reaching only CW 20 mm.

**Table 2.** Morphometric and collection data of specimens of *Louiseayabassi* from Ebo Forest, Cameroon, and *L.nkongsamba* from Nlonako Ecological Reserve, Cameroon. Ad: adult; Sa: subadult, M: male; F: female.

Species	CW/FW mean (*n*)	CL/FW mean (*n*)	CH/FW mean (*n*)	Size range (CW in mm)	Museum number	Locality	Geographic coordinates	Altitude (m a.s.l.)
*L.yabassi*	2.9 (19)	2.1 (19)	1.3 (19)	Ad M 16.4–20.2	LaboPasmat X100	Ebo Forest, Stream NO. 1	04°25'01.7"N, 010°12'00.8"E	162
*L.yabassi*				Ad F 12.0–24.1	ZMB Crust 33829	Ebo Forest, Stream NO. 1	04°25'01.7"N, 10°12'00.8"E	162
*L.yabassi*	2.9 (16)	2.1 (16)	1.3 (16)	Ad M 16.6–21.3	LaboPasmat X101	Ebo Forest, Stream NO. 2	04°24'59.3"N, 010°12'07.7"E	254
*L.yabassi*				Ad F 17.4–22.5	ZMB Crust.33775	Ebo Forest, Stream NO. 2	04°24'59.3"N, 010°12'07.7"E	254
*L.nkongsamba*	2.9 (8)	2.1 (8)	1.3 (8)	Ad M 15.8–20.0	LaboPasmat X102	Nlonako, Nguengue	04°54'44.8"N, 009°58'50.0"E	1176
*L.nkongsamba*	2.9 (5)	2.1 (5)	1.3 (5)	Ad M 12.8–18.5	LaboPasmat X102Y	Nlonako, NgaltongueS2	04°55'20.4"N, 009°57'31.0"E	1180
*L.nkongsamba*	2.9 (12)	2.1 (12)	1.3 (12)	Ad M 13.8–17.4	LaboPasmat X103	Nlonako, NgaltongueS1	04°55'20.4"N, 009°57'42.6"E	1180
*L.nkongsamba*	2.9 (10)	2.1 (10)	1.3 (10)	Sa M 11.7–11.8	ZMB Crust.33789	Nlonako, Engugue1382	04°54'21.6"N, 009°58'20.6"E	1382
*L.nkongsamba*	2.9 (11)	2.1 (11)	1.3 (11)	Sa M 11.5–14.4	LaboPasmat X104	Nlonako, Eyimba	04°53'30.7"N, 009°59'05.1"E	1194
*L.nkongsamba*	2.9 (6)	2.1 (6)	1.3 (6)	Ad 12	LaboPasmat X104Y	Nlonako, Engugue1462	04°54'21.9"N, 009°58'22.4"E	1462
*L.nkongsamba*				Ad F 14–15	LaboPasmat X105	Nlonako, Engugue1462	04°54'21.9"N, 009°58'22.4"E	1462
*L.nkongsamba*				Sa 6.60	LaboPasmat X105Y	Nlonako, Engugue1462	04°54'21.9"N, 009°58'22.4"E	1462
*L.edeaensis*	3.0 (21)	2.5 (21)	1.4 (21)	Ad M 14.1–17.5	See Mvogo Ndongo et al. 2019: 143, table 2	Lake Ossa	03°48'56.1"N, 010°03'18.5"E	90
*L.edeaensis*				Ad F 13.0–19.9	See Mvogo Ndongo et al. 2019: 143, table 2	Lake Ossa	03°48'56.1"N, 010°03'18.5"E	90
*L.balssi*	2.9 (8)	2.1 (8)	1.2 (8)	Ad M 13.3–16.2	See Mvogo Ndongo et al. 2019: 147, table 3	Manengouba	05°01'56.9"N, 009°49'37.8"E	1958
*L.balssi*				Ad F 13.3–14.8	See Mvogo Ndongo et al. 2019: 147, table 3	Manengouba	05°01'56.9"N, 009°49'37.8"E	1958

**Figure 2.**
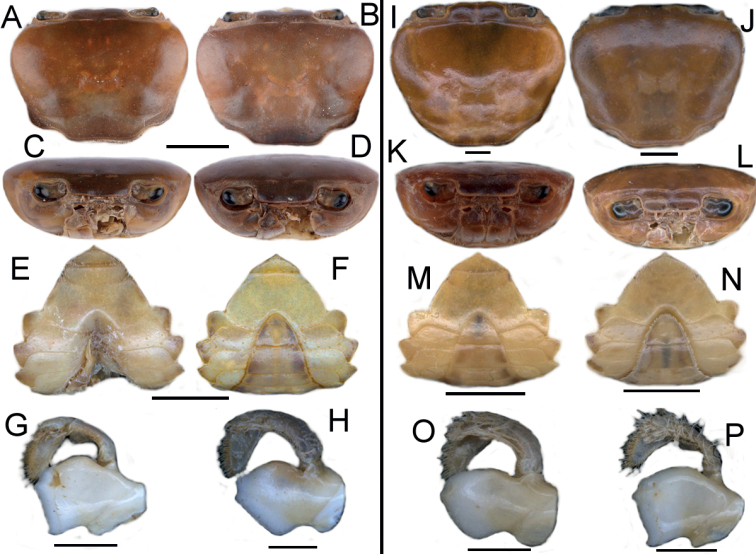
*Louiseayabassi* from Ebo Forest, Cameroon, adult male (CW 20.2 mm) from site no. 1 (**A, C, E, G**), adult male (CW 21.3 mm) from site no. 2 (**B, D, F, H**). *Louiseankongsamba* from Nlonako, Cameroon, adult male (CW 18.2 mm) from Eyimba (**I, K, M, O**), subadult male (CW 12.0 mm) from Enguegue (site no. 1) (**J, L, M, P**). **A, B, I, J** dorsal view of cephalothorax **C, D, K, L** frontal view of cephalothorax **E, F, M, N** ventral view of thoracic sternum **G, H, O, P** frontal view of left mandible. Scale bars: 8 mm (**A, C, E**); 9 mm (**B, D, F**); 1 mm (**G, H**); 4 mm (**I, K**); 12 mm (**J, M**); 3 mm (**L**); 8 mm (**N**); 2 mm (**O, P**).

**Figure 3.**
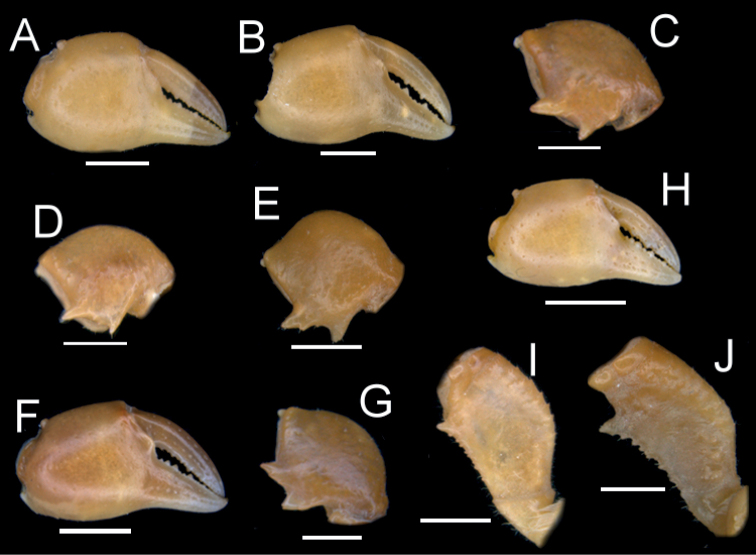
*Louiseankongsamba* from Nlonako, Cameroon, subadult male (CW 12.0 mm) from Enguegue (site no. 1) (**A, D, I**), adult male (CW 18.2 mm) from Eyimba (**B, E, J**). *Louiseayabassi* from Ebo Forest, Cameroon, adult male (CW 21.3 mm) from site no. 2 (**C, H**), adult male (CW 20.2 mm) from site no. 1 (**F, G**). **A, B, F, H** frontal view of chela **C, D, E, G** cheliped carpus **I, J** cheliped merus. Scale bars: 5 mm (**A–J**).

**Figure 4.**
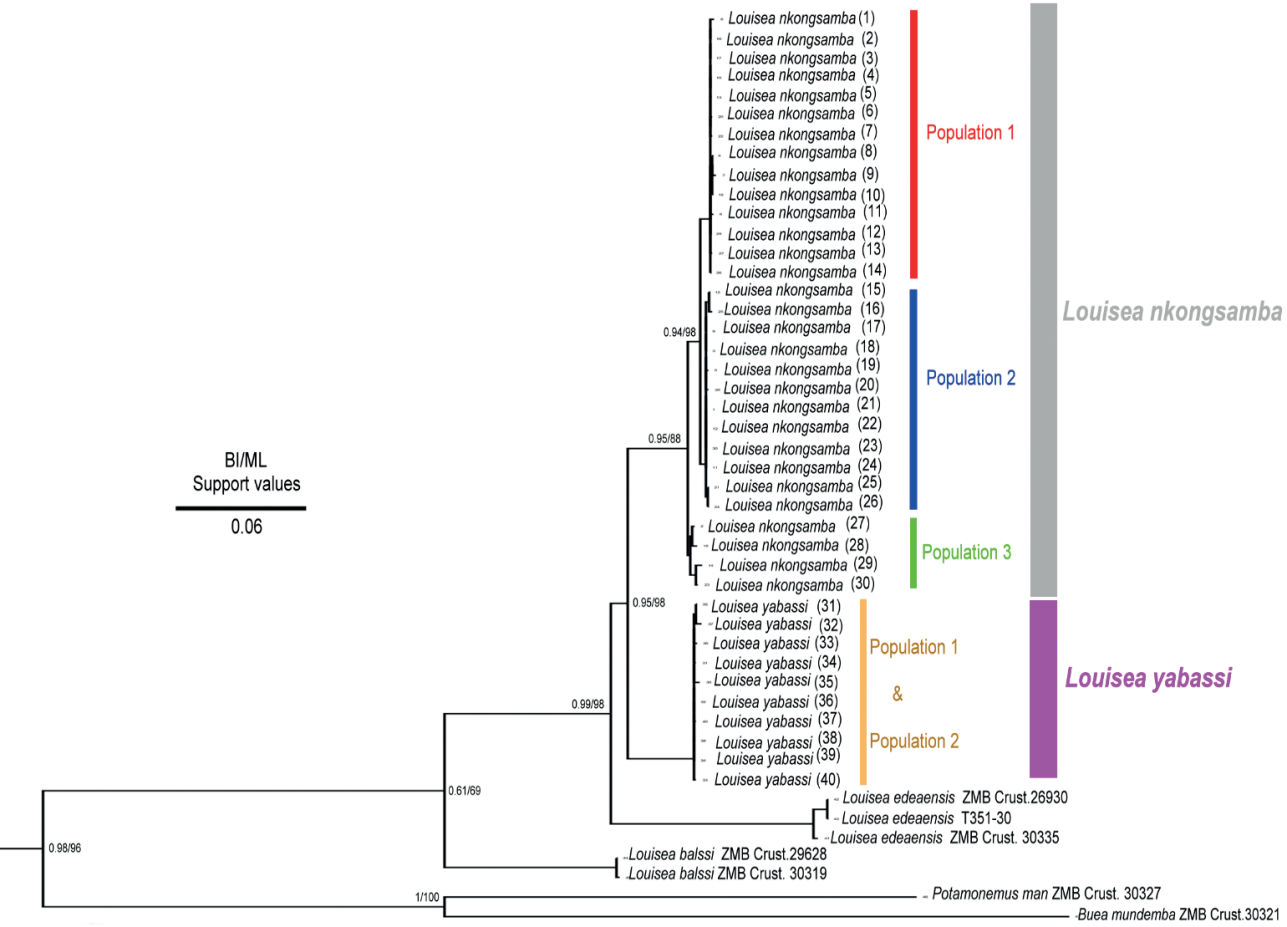
ML tree topology for *Louisea* species of Cameroon, derived from mtDNA sequences corresponding to three mtDNA loci (partial 12S rRNA, 16S rRNA, and COI genes). BI and ML statistical values (%) on the nodes indicate posterior probabilities and bootstrap support, respectively.

**Figure 5.**
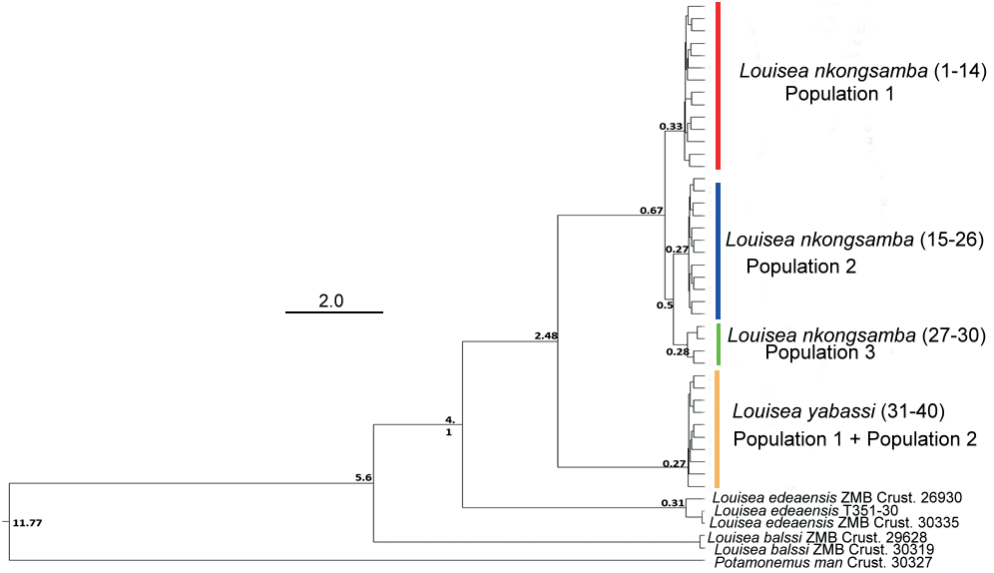
BI tree topology for *Louisea* species of Cameroon, derived from COI mtDNA sequences. Statistical values on the nodes indicate dates in millions of years.

**Figure 6.**
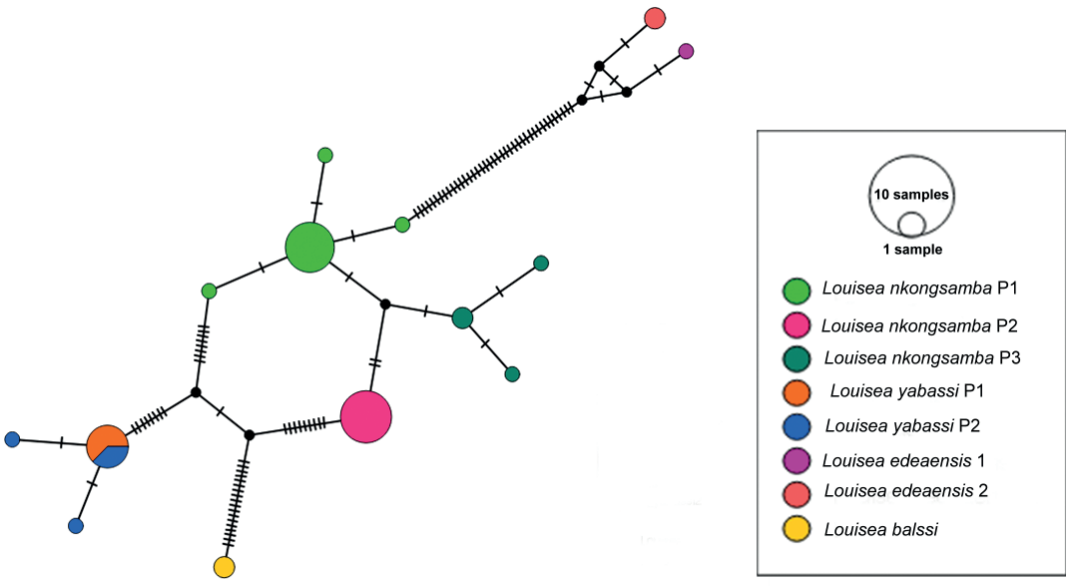
Maximum parsimony genotype networks for *Louisea* species of Cameroon, derived from COI mtDNA sequences. Hatch marks stand for mutation steps.

Differences in certain morphological characters of the specimens of *L.yabassi* from two populations in the Ebo Forest are noteworthy (Table 3). Like *L.yabassi*, *L.nkongsamba* also showed differences in several morphological characters among the specimens from six sites, which are organised here into morphotype 1 (Nlonako Enguegue NO. 1_1462) and morphotype 2 (Nlonako Eyimba, Ngaltongue, Enguegue NO. 2_1382 m, Nguegue) (Table 4). These morphological differences between the two populations/morphotypes of *L.yabassi* and *L.nkongsamba* are also illustrated (Figs 2, 3). Despite those morphological differences, there is no genetic support for recognising these differences as indicating different genetic lineages that would warrant formal taxonomic recognition (Figs 4–6).

**Table 3.** Comparison of selected morphological characters between two populations (morphotypes) of *Louiseayabassi* from Ebo Forest, Cameroon.

Character	Population no. 1 (morphotype 1)	Population no. 2 (morphotype 2)
Epibranchial tooth	reduced to granule (Fig. 2A, C)	small (Fig. 2B, D)
Intermediate tooth between exorbital & epibranchial teeth	distinct, but small (Fig. 2A, C)	relatively large, triangular (Fig. 2B, D)
Major cheliped dactylus	slim, gently arched (Fig. 3F)	slim, almost straight (Fig. 3H)
Cheliped carpus inner margin teeth	both distal and proximal teeth large, positioned some distance from each other (Fig. 3G)	distal tooth larger than proximal tooth, positioned relatively closer to each other (Fig. 3C)
Mandible inferior lateral corner of coxa (biting edge)	lacking pointed tip (Fig. 2G)	with pointed tip (Fig. 2G)
Margin of male sternal sulcus S3	with long setae (Fig. 2E)	lacking setae (Fig. 2F)
Male sternal sulcus S3/4	reduced to 2 deep lateral notches (Fig. 2E)	indiscernible (Fig. 2F)

**Table 4.** Comparison of selected morphological characters between two populations (morphotypes) of *Louiseankongsamba* from Mount Nlonako, Cameroon.

Characters	Morphotype 1	Morphotype 2
	Nlonako Engugue1462	Nlonako Eyimba, Ngaltongue, Engugue1382, Nguegue and type specimens
Exorbital tooth	relatively large (Fig. 2J, L)	relatively small (Fig. 2I, K)
Epibranchial tooth	small (Fig. 2J, L)	reduced to granule (Fig. 2I, K)
Intermediate tooth between exorbital & epibranchial teeth	relatively large (Fig. 2J, L)	relatively small (Fig. 2I, K)
Lateral margin posterior to epibranchial tooth	lined with small granules (Fig. 2J)	smooth (Fig. 2I)
Postfrontal crest	poorly defined, completely traversing carapace, reaching anterolateral margins at intermediate tooth (Fig. 2J, L)	clearly defined, completely traversing carapace, not reaching anterolateral margins (Fig. 2I, K)
Major cheliped dactylus	slim, straight (Fig. 3A)	slim, gently arched (Fig. 3B)
Cheliped carpus inner margin teeth	distal tooth larger than proximal tooth, both slender, positioned some distance from each other (Fig. 3D)	distal tooth larger than proximal tooth, both robust, positioned relatively closer to each other (Fig. 3E)
Medial inferior margin of cheliped merus	with small but distinct jagged distal tooth angled outward at 60°, followed by numerous granules and small teeth (Fig. 3I)	with large jagged distal tooth angled outward at 90°, followed by numerous granules and small teeth decreasing in size proximally (Fig. 3J)
Mandible inferior lateral corner of coxa (biting edge)	lacking pointed tip (Fig. 2P)	with pointed tip (Fig. 2O)
Male sternal sulcus S3/4	indiscernible except for 2 deep lateral notches (Fig. 2N)	indiscernible, lacking lateral notches (Fig. 2M)

The pubertal moult estimates indicate that the largest *Louisea* species is *L.yabassi* (CW 24 mm); the smallest species is *L.balssi* (CW 16.2 mm); while the size ranges of *L.edeaensis* and *L.nkongsamba* overlap with each other (~CW 20 mm) in between those of *L.balssi* and *L.yabassi* (Table 2). *Louiseabalssi* is a high-altitude species that dwells at 1958 m a.s.l.; *L.nkongsamba* is a submontane species found between 938 and 1462 m a.s.l.; while both *L.edeaensis* and *L.yabassi* are low-altitude crabs, occurring at 90 m a.s.l. and 300 m a.s.l., respectively (see Mvogo Ndongo et al. 2017a, 2017c, 2019, 2021b; Table 2).

### ﻿Molecular analyses

The present molecular analyses support recognition of three lineages (as population 1, 2, and 3) of *L.nkongsamba* from six sites on Mount Nlonako (Figs 4–6). These distinct lineages, however, do not correlate with the two morphotypes recognised herein for *L.nkongsamba* (Table 4). Population 1 of *L.nkongsamba* included specimens that were collected from all six localities of Mount Nlonako (Tables 1, 5; Fig. 4); population 2 of *L.nkongsamba* comprised specimens that were collected from five out of six sites of Mount Nlonako (Tables 1, 5; Fig. 4); and population 3 of *L.nkongsamba* comprised specimens that were collected from four out of six sites of Mount Nlonako (Tables 1, 5; Fig. 4). Both morphotypes of *L.nkongsamba* are represented in one or the other population (Table 1).

**Table 5.** Number of individuals of *Louiseankongsamba* studied per site/population.

Sites	Altitude (m a.s.l.)	Number of individuals in Population 1	Number of individuals in Population 2	Number of individuals in Population 3
Enguegue no. 2	1382	1	3	1
Ngaltongue no. 1	1176	4	1	0
Ngaltongue no. 2	1256	4	0	1
Enguegue no. 1	1462	1	3	1
Nguegue	1211	3	2	0
Eyimba	938	1	3	1
**Total**		14	12	4

The uncorrected *p*-distance between *Louisea* species pairs reveal that each is well isolated from other taxa assigned to this genus (Table 6). *Louiseankongsamba* is sister species to *L.yabassi* with relatively low *p*-distance (3.97%) (Table 6); both are sister to *L.edeaensis*. *Louiseabalssi* is isolated from *L.edeaensis*, with a sequence divergence of 11.04% (12S rRNA), 10.15% (COI), and 7.77% (16S rRNA) (Table 6); from *L.yabassi*, with a sequence divergence of 12.94% (12S rRNA), 7.32% (COI), and 5.36% (16S rRNA); and from *L.nkongsamba*, with a sequence divergence of 12.42% (12S rRNA), 7.98% (COI), and 5.04% (16S rRNA) (Table 6). The uncorrected *p*-distances between the three genetic populations of *L.nkongsamba* are given in Table 7. Population 1 of *L.nkongsamba* is sister to population 2, both populations are sister to population 3.

**Table 6.** Pairwise uncorrected *p*-distances of COI, 16S rRNA, and 12S rRNA partial sequences between the species of *Louisea*.

*Louisea* species	Uncorrected *p*-distance		
	COI	16S rRNA	12S rRNA
*L.nkongsamba* and *L.yabassi*	3.97%	2.15%	3.77%
*L.nkongsamba* and *L.edeaensis*	8.61%	4.33%	4.92%
*L.nkongsamba* and *L.balssi*	7.98%	5.04%	12.42%
*L.edeaensis* and *L.yabassi*	8.88%	4.35%	4.27%
*L.edeaensis* and *L.balssi*	10.15%	7.77%	11.04%
*L.yabassi* and *L.balssi*	7.32%	5.36%	12.94%

The phylogenetic analysis indicates that *L.balssi* from Mount Manengouba is the ancestral species, while *L.edeaensis* from Lake Ossa is the sister species of the clade that includes *L.yabassi* and *L.nkongsamba* (Fig. 4). Divergence time calculations of *Louisea* species (Fig. 5) showed that the early divergence within the genus occurred during the late Miocene, i.e., *L.balssi* diverged from other species at about 5.6 myr. *Louiseayabassi* and *L.nkongsamba* diverged from *L.edeaensis* at about 4.1 myr, and *L.yabassi* separated from *L.nkongsamba* at about 2.48 myr.

The haplotype network recovered eight haplotypes for *L.nkongsamba* with maximum four mutation steps between the specimens of this species (Fig. 6) and distinguishes between the four *Louisea* species (Fig. 6).

## ﻿Discussion

### ﻿Phylogenetic and phylogeographic relationships

The four *Louisea* species recovered here each has a monophyletic clade (Fig. 4) with strong topological statistical support, and high pairwise uncorrected *p*-distance values between species pairs (Table 6). This study, therefore, supports the continued recognition of all four *Louisea* species that are endemic to the southwest Cameroon rainforests. The earliest divergence for *Louisea* species happened at ~5.6 myr (late Miocene; Fig. 5), which corresponds to the dates for cladogenesis within genera provided by Daniels et al. (2015). In contrast, the latest *Louisea* divergence between *L.nkongsamba* and *L.yabassi* seems to have occurred during the late Pliocene (2.48 myr). Similar divergence times were recovered for another Central African freshwater crab species pair, *Sudanonautesaubryi* (H. Milne Edwards, 1853) and *S.floweri* (De Man, 1901) (see Daniels et al. 2015: fig. 3). Even the two morphologically variable *Louisea* species, *L.nkongsamba* and *L.yabassi*, were found in the molecular analyses to have low uncorrected *p*-distance values (Table 6), but both are recognised as distinct (see Mvogo Ndongo et al. 2019).

**Table 7.** Pairwise uncorrected *p*-distances of COI, 16S rRNA, and 12S rRNA partial sequences between the populations of *Louiseankongsamba*.

*Louiseankongsamba*	Uncorrected *p*-distance		
	COI	16S rRNA	12S rRNA
Population 2 and Population 3	0.48%	0.87%	0.95%
Population 2 and Population 1	0.70%	0.20%	0.71%
Population 3 and Population 1	0.52%	0.61%	1.18%

*Louisea* species are found in different habitats within the rainforest zone: *L.balssi* in montane forest streams; *L.nkongsamba* in submontane forest streams; *L.edeaensis* on the islands of a freshwater lake; and *L.yabassi* in lowland forest streams. *Louiseankongsamba* specimens from cool mountain streams draining the submontane forests of Mt. Nlonako (938–1462 m a.s.l.) are small-bodied with adult males measuring CWs 16–20 mm. *Louiseabalssi* adult males from the cool high-altitude streams (1,958 m a.s.l.) draining into the caldera of Mount Manengouba are also noticeably small-bodied (CWs 13.0–16.2 mm). This agrees with the findings of Daniels et al. (2016) who reported that genetic differentiation tends to be somewhat limited in small-bodied montane species of freshwater crabs. Only a limited genetic variation, however, was found in the lowland forest species, *L.edeaensis*. In comparison, the moist tropical rainforests surrounding Mount Manengouba receive a high annual rainfall that has maintained a stable forest ecosystem, even during drier periods in the past when rain forests were replaced by savannas in other parts of Central Africa (Brown and Ab’Saber 1979; Diamond and Hamilton 1980; Mayr and O’Hara 1986; Grubb 1992; Zimkus 2009). Consequently, in such high rainfall areas, *L.balssi* would be sheltered from the harsher effects of rainforest disruption arising from prolonged dry periods in the past, making the Cameroon Highlands a Pleistocene forest refuge for freshwater crab species. Over time, *Louisea* dispersed from its original location around Mount Manengouba into the surrounding forests of southwest Cameroon, including Mount Nlonako. There *L.nkongsamba* evolved and continued to disperse into the forested lowlands around Yabassi and Lake Ossa, where *L.yabassi* and *L.edeaensis* evolved.

### ﻿Intraspecific morphological variation

The two *L.yabassi* populations from localities ~2–3 km apart in the Ebo Forest genetically form a single clade with little lineage differentiation (Fig. 4; populations 1 and 2), and these individuals show relatively low levels of morphological variation (Table 3). Despite this, two *L.yabassi* morphotypes could be identified (Table 3). Similarly, the six sampled localities around Mount Nlonako, where *L.nkongsamba* is found, are 4–10 km apart (Tables 2, 5). These individuals of *L.nkongsamba* fall into three genetically recognisable populations (Fig. 4; populations 1–3), which in turn have two distinct morphotypes (Table 4). Populations 1 and 3 consisted of individuals that all belong to morphotype 1, while population 2 included individuals of both morphotypes (Table 1). The high carapace (CH/FW = 1.3) and narrow front width (CW/FW = 2.9) of both *L.yabassi* and *L.nkongsamba* are associated with a semi-terrestrial, air-breathing lifestyle (Cumberlidge 1999). Populations of both species prefer temporary water bodies such as puddles near small permanent streams, as well as damp environments under small stones or in forest floor leaf litter adjacent to streams (Mvogo Ndongo et al. 2017a, 2018, 2019, 2021b). Freshwater crabs have limited dispersal abilities due to the absence of a free-swimming larval phase and their direct development resulting in crab hatchlings; the limited dispersal abilities of the crabs and the restricted movements of the adults in combination with the isolated and fragmentary nature of their wetland habitats might be at least partly responsible for their rich diversity and high endemism (Cumberlidge et al. 2009; Mvogo Ndongo et al. 2021b). The intraspecific morphological and genetic variations observed within *L.yabassi* and *L.nkongsamba* are crucial for adaptation by natural selection, not least because low levels of variation are associated with the extirpation of populations and an increased risk of species extinction (Bolnick et al. 2011; Scheiner and Holt 2012; Forsman 2014).
